# Increased expression of advanced glycation endproducts in the gingival crevicular fluid compromises periodontal status in cigarette-smokers and waterpipe users

**DOI:** 10.1186/s12903-022-02240-z

**Published:** 2022-05-25

**Authors:** Dena Ali, Fatemah AlAhmari, Toshinari Mikami, Jagan Kumar Baskaradoss

**Affiliations:** 1 Department of General Dental Practice, Kuwait University, P. O. Box 24923, 13110 Safat, Kuwait; 2 Department of Periodontics and Community Dentistry, College of Dentistry, King Saud University, Riyadh, Saudi Arabia; 3 Pax Creation Medical Lab, Morioka, Japan; 4 Department of Oral Pathology, Oral Lab Central College of Stomatology, China Medical University, Shenyang, China; 5 Department of Developmental and Preventive Sciences, Kuwait University, Kuwait City, Kuwait

**Keywords:** Alveolar bone loss, Advanced glycation endproducts, Clinical attachment loss, Plaque index, Smoking

## Abstract

**Background:**

The aim was to assess the association between levels of advanced glycation endproducts (AGEs) in the gingival crevicular fluid (GCF) and periodontal parameters among cigarette-smokers and waterpipe-users.

**Methods:**

Self-reported cigarette-smokers; waterpipe-users and never-smokers were included. Demographic data was recorded using a questionnaire. Periodontal parameters (plaque index [PI], gingival index [GI], clinical attachment loss [AL], probing depth [PD], and marginal bone loss [MBL]) were assessed in all groups. The GCF samples were collected using standard techniques and assessed for AGEs levels using enzyme-linked immunosorbent assay. Sample-size estimation was done and group-comparisons were done. Correlation between levels of GCF AGEs levels and periodontal parameters was assessed using a logistic regression model. Level of significance was set at *P* < 0.01.

**Results:**

Eighty-two individuals (28 cigarette-smokers, 28 waterpipe-users and 26 never-smokers) were included. There was no difference in mean ages of all patients. Cigarette-smokers had a smoking history of 5.1 ± 0.2 pack years and waterpipe-users were using waterpipe for 4.4 ± 0.6 years. There was no statistically significant difference in PI, GI, clinical AL, PD and MBL in all groups. Levels of AGEs were significantly higher among cigarette-smokers (*P* < 0.001) and waterpipe-users (*P* < 0.001) than never-smokers. There was no significant correlation between levels of GCF AGEs levels and periodontal parameters in all groups.

**Conclusion:**

Clinical periodontal status of individuals with a short history of cigarette-smoking and waterpipe-usage may appear similar to never-smokers. On a molecular level, cigarette-smoking and waterpipe-users express raised levels of AGEs than never-smokers that sirens about the ongoing yet latent periodontal inflammatory process.

## Introduction

The pathophysiology of periodontal inflammatory conditions in susceptible patient groups is complex as a variety of genetic and immunoinflammatory mechanisms have been reported [1–6]. Recent studies [1, 2, 7] have shown that raised salivary levels of inflammatory proteins including matrix metalloproteinases-9 (MMP-9), tumor necrosis factor-alpha (TNF-α) and nod-like receptor family pyrin domain-containing protein-3 (NLRP3) complex inflammasome contribute towards the occurrence of periodontitis. Similarly, the gingival crevicular fluid (GCF) is another biological fluid that expresses raised levels of inflammatory proteins such as TNF-α and advanced glycation endproducts (AGEs) in patients with periodontal diseases [8, 9]. The AGEs are glycotoxins and are highly oxidant compounds with a pathogenic significance [10]. Studies [11–13] have shown that interaction between AGEs and their receptors (RAGE) induce a state of oxidative stress in tissues including those of the periodontium.

Habitual use of combustible tobacco products such as cigarettes is a well-known risk factor of oral diseases including periodontitis [14–17]. From a clinical perspective, studies [18, 19] have shown that cigarette-smoking as well as waterpipe usage are risk-factors of periodontitis as these individuals demonstrate significantly high scores of plaque index (PI), probing depth (PD), clinical attachment loss (AL), and radiologic marginal bone loss (MBL) compared with never-smokers. Laboratory-based investigations [10, 20] have also shown that tobacco-smoking is associated with increased formation and accumulation of AGEs in periodontal tissues compared with never-smokers. In the study by Katz et al. [10], biopsies of gingival epithelial cells (GECs) were taken from five smokers and were compared with sex- and age-matched controls (never-smokers) for the expression of RAGE. The results showed that nicotine elevates the expression of RAGE in GECs, which reflects that AGEs-RAGE communications are linked with the etiopathogenesis of periodontitis in smokers [10]. Waterpipe (also known as narghile, hubble-bubble or/and hookah) is another form of tobacco-smoking, which is a cultural norm in many Middle-Eastern countries such as Kuwait and Saudi Arabia [21–24]. However, habitual use of waterpipe is now prevalent in many European countries and the United States [25–27]. Waterpipe users often perceive that this form of tobacco inhalation is less injurious to health compared with conventional cigarette-smoking [28]. Nevertheless, scientific evidence has also shown that the health-hazards of waterpipe-use are similar to those of cigarette-smoking [29]; and waterpipe-usage is “a global public health problem” [25]. Mokeem et al. [30] reported that clinical and radiographic parameters of periodontal inflammation are poorer in cigarette-smokers and waterpipe-users than never-smokers with no statistically significant difference between the former groups (cigarette-smokers and waterpipe-users). However, from an immunoinflammatory perspective, there are no studies that have compared GCF AGEs levels among cigarette-smokers, waterpipe users and never-smokers. The authors hypothesize that the levels of AGEs are significantly high in GCF of cigarette-smokers and waterpipe-users compared with never-smokers.

The aim was to assess the association between levels of AGEs in the GCF and periodontal parameters among cigarette-smokers and waterpipe-users.

## Methods

### Inclusion and exclusion criteria

The inclusion criteria were as follows: (a) Self-reported systemically healthy adults (males and females aged at least 18 years and older); (b) self-reported current cigarette-smokers (individuals who were currently smoking and smoked at least 1 cigarette daily for ≥ 12-months [31]); (b) self-reported waterpipe-users (individuals who had used waterpipe at least once within the last 4-weeks [32]); and (c) self-reported never-smokers (individuals who reported to have never used any form of tobacco-product [33]). The following criteria were used for exclusion: (a) dual-smokers (individuals smoking cigarettes and simultaneously using other forms of nicotinic products such as electronic nicotine delivery systems, waterpipe, *bidis*, pipe, cigar etc.); (b) pregnancy and/or lactation; and (c) individuals that reported to have used antibiotics, anti-inflammatory medications, bisphosphonates, probiotics and/or cancer therapy. Grossly-carious and supernumerary teeth and third molars were not assessed.

### Grouping

Individuals that volunteered to participate in the present study were categorized into 3 groups as follows: Group-1 comprised of self-reported cigarette-smokers; Group-2 comprised of self-reported waterpipe-users; and in Group-3 self-reported never-smokers were included.

### Questionnaire

A questionnaire printed in simple Arabic and English with open-ended questions was presented to all participants. Data regarding gender, age in years, duration of cigarette-smoking habit, number of cigarette/packs smoked daily, duration of waterpipe smoking habit, number of times waterpipe was smoked every day, duration (in minutes) of each session of waterpipe usage, number of puffs per session of waterpipe usage and daily toothbrushing and interdental flossing was recorded by one investigator (DA).

### Collection of gingival crevicular fluid

Sterile cotton rolls were placed in the buccal and lingual sulci of the mandibular right first molar tooth and air-dried. Supragingival plaque was gently eradicated using sterile curettes; and a sterile paper-strip (Periopaper®, Interstate Drug Exchange, Amityville, NY, USA) was inserted in the mid-buccal sulcus until resistance was felt. The paper strip (Periopaper®, Interstate Drug Exchange, Amityville, NY, USA) was held in place for 10 s. Volume of the collected GCF was immediately measured using an electronic calibrated machine (Periotron 8000, Oraflow. Inc., Hewlett, NY, USA). The sample was pooled and eluted in microcentrifuge tubes containing 400 µl phosphate buffered saline for 60 min prior to freezing at -80ºC. Samples contaminated with saliva and/or blood were discarded and fresh GCF samples from the same site were re-collected after a waiting period of 30 min.

### Analysis of AGEs in GCF

All GCF samples were centrifuged at 5000×*g* for 20 min in a cold room. Aliquots of each sample were assayed by enzymatic immunosorbent assay to determine the levels of AGEs using an assay kit (abcam AGE Assay kit—ab238539, Cambridge, MA 02139–1517, USA), which was used as per the manufacturer’s instructions. The sensitivity of the assay kit was 0.5 µg/ml. All samples were assessed in duplicates. The enzyme reaction was stopped by adding 100 µL of Stop Solution. Absorbance was read immediately on a microplate reader at 450 nm; and amounts of AGEs were recorded in micrograms per milliliter (µg/ml).

### Periodontal parameters

Clinical periodontal assessment was done 24 h after GCF sample collection by a calibrated investigator (JKB; Kappa score 0.88) who was blinded to the study-groups. Full-mouth plaque index PI [34], gingival index (GI) [35], were assessed at the buccal, lingual/palatal, mesial, and distal sites on all teeth. Clinical AL [36] and PD [37] were measured to the nearest millimeter with a graded probe (Hu-Friedy InC, Chicago, IL, USA) at the mesiobuccal, midbuccal, distobuccal, distolingual/palatal, midlingual/palatal and mesiolingual/palatal sites. Full-mouth digital intra-oral radiographs (Planmeca Romexis Intra oral X-Ray, Planmeca OY, Helsinki, Finland) were taken [6]; and standardization of all x-rays was done as described elsewhere [38, 39]. All radiographs were examined by one researcher (DA; Kappa score 0.86). Numbers of missing teeth were counted and recorded.

### Statistical and power analyses

Quantitative analysis was done using the one-way-analysis-of-variance and Bonferroni post-hoc correction tests (SPSS., V20, Chicago,IL, USA). Data normality was determined via Kolmogrov-smirnov test. A Probability value that was less than 0.01 was selected as a marker of statistical-significance. Prior sample-size estimation was done using data obtained from a pilot investigation (nQuery Advisor/5; Statistical-Solutions, Saugus.M.A, USA). Sample-size estimation was based on the presumption that a mean difference of 1 mm in clinical-AL and PD should be detected at a significance-level of 0.01 and a desired study power of at least 80%. With inclusion of 26 patients per group, the present investigation was projected to achieve 90% power with a 1% two-sided significance-level.

## Results

### Demographic characteristics

Twenty-eight, 28 and 26 cigarette-smokers, waterpipe-users and never-smokers, respectively were included. Twenty, 25 and 21 cigarette-smokers, waterpipe-users and never-smokers, respectively were male. There was no significant difference in the mean ages of individuals in all groups. Cigarette-smokers were smoking 0.8 ± 0.2 packs daily and had a smoking history of 5.1 ± 0.2 pack years. Waterpipe-users were using the hubble-bubble for 4.4 ± 0.6 years and were using waterpipe 7.5 ± 0.3 times daily. The mean duration of each session of waterpipe usage was 15.5 ± 3.1 min and were inhaling 12.2 ± 0.4 puffs per session. Toothbrushing twice daily was reported by 92.9% cigarette-smokers 89.3% waterpipe-users and 96.2% never-smokers. Interproximal flossing once daily was reported by 71.4% cigarette-smokers, 64.3% waterpipe-users and 73.1% never-smokers (Table 1).

**Table 1:** Demographic parameters of the study groups

Parameters	Cigarette-smokers	Waterpipe-users	Never-smokers (controls)
Number of patients	28	28	26
Gender (male)	20	25	21
Mean age (all patients)	29.6 ± 1.2 years	27.4 ± 0.8 years	26.8 ± 0.5 years
Duration of smoking habit in years	6.4 ± 1.2 years	NA	NA
Number of cigarette packs smoked daily	0.8 ± 0.2 packs/day	NA	NA
Pack-years	5.1 ± 0.2 pack years	NA	NA
Duration of waterpipe-usage in years	NA	4.4 ± 0.6 years	NA
Daily waterpipe usage (number of times daily)	NA	7.5 ± 0.3 times daily	NA
Duration in minutes of each session of waterpipe usage	NA	15.5 ± 3.1 min	NA
Number of puffs per session of waterpipe usage	NA	12.2 ± 0.4 puffs per session	NA
*Daily tooth-brushing*			
Once daily (n) (%)	2 (7.1%)	3 (10.7%)	1 (3.8%)
Twice daily (n) (%)	26 (92.9%)	25 (89.3%)	25 (96.2%)
*Daily flossing*			
Once daily (n) (%)	20 (71.4%)	18 (64.3%)	19 (73.1%)
Twice daily (n) (%)	None	None	None
Never	8 (28.6%)	10 (35.7%)	7 (26.9%)

### Periodontal parameters

Cigarette-smokers, waterpipe-users and never-smokers had 5, 6 and 3 missing teeth, respectively. There was no significant difference in P, GI, PD, clinical AL and MBL among cigarette-smokers, waterpipe-users and never-smokers (Table 2).

**Table 2:** Periodontal parameters of the study groups

Parameters	Cigarette-smokers	Waterpipe-users	Controls
Number of missing teeth	5	6	3
Plaque index^*^	0.7 ± 0.005	0.6 ± 0.07	0.6 ± 0.05
Gingival index^†^	0.6 ± 0.08	0.7 ± 0.004	0.5 ± 0.003
Clinical attachment loss	0.4 ± 0.04	0.5 ± 0.1	0.3 ± 0.003
Probing depth	0.7 ± 0.04	0.8 ± 0.06	0.5 ± 0.1
Marginal bone loss (mesial)	0.8 ± 0.05 mm	1.01 ± 0.04 mm	0.5 ± 0.04 mm
Marginal bone loss (distal)	0.9 ± 0.005 mm	1.02 ± 0.08 mm	0.4 ± 0.003 mm

*Measured at four sites per tooth; †Measured at four sites per tooth

### GCF Volume and levels of AGEs

The volume of collected GCF was significantly higher among cigarette-smokes (*P* < 0.001) and waterpipe-users (*P* < 0.001) compared with never-smokers. There was no significant difference in the collected GCF volume among cigarette-smokes and waterpipe-users. The GCF AGEs levels were significantly higher among cigarette-smokes (*P* < 0.001) and waterpipe-users (*P* < 0.001) compared with never-smokers. There was no significant difference in the collected GCF volume among cigarette-smokes and waterpipe-users (Table 3).

**Table 3:** Volume of gingival crevicular fluid and concentrations of advanced glycation endproducts in the study groups

Parameters	Cigarette-smokers	Waterpipe-users	Controls
GCF (in µl)	0.82 ± 0.4 µl*	0.73 ± 0.3 µl*	0.3 ± 0.02 µl
AGEs concentration (µg/ml)	225.4 ± 29.7 µg/ml*	216.6 ± 16.5 µg/ml*	69.6 ± 3.2 µg/ml

*Compared with controls (*P* < 0.001)

### Correlation of AGEs with clinical, radiographic and demographic parameters

The GCF AGES levels showed no statistically significant correlation between PD and clinical AL (Table 4). In all groups, no correlation existed between GCF AGEs levels and patients’ age, gender, PI, GI, and MBL (data not shown).

**Table 4:** Correlation of probing depth and clinical attachment loss with advanced glycation endproducts in cigarette-smokers, waterpipe users and never-smokers

Groups	Confidence interval	R.^2^	*P*-value
*Probing depth*			
Cigarette-smokers	− 80.9 to 85.4	0.0018	0.95
Waterpipe-users	− 28.03 to 68.7	0.027	0.40
Controls	− 69.2 to 15.44	0.06	0.21
*Clinical attachment loss*			
Cigarette-smokers	− 34.5 to 55.6	0.421	0.19
Waterpipe-users	− 19.6 to 32.1	0.524	0.25
Controls	− 25.2 to 37.6	0.414	0.19

## Discussion

The present study was based on the hypothesis that levels of AGEs are significantly high in GCF of cigarette-smokers and waterpipe-users compared with never-smokers. In other words, the authors of the present study expected that scores of PI, PD, clinical AL and MBL would be markedly higher among cigarette-smokers and waterpipe-users compared with never-smokers. Surprisingly, the results showed otherwise as the clinico-radiographic outcomes showed no statistically significant difference in all groups. There are several factors that may have contributed with such a finding. Firstly, all individuals that agreed to participate in the present study were young (approximately 30 years old); however, all individuals that agreed to participate and sign the written informed consent form were young (age range 23–32 years). It is pertinent to mention that in the present study, there were no stringent criteria to include individuals belonging to a particular age group in the present study. Another potential factor is a short history of tobacco usage among patients in groups 1 and 2. Based upon these results, cigarette-smokers may be considered “mild cigarette-smokers) as they had a smoking history of approximately 5 pack-years. Similarly, water-pipe users were adapted with this habit for nearly 4.5 years. With regards to routine oral hygiene maintenance, the present results suggest that the patient population investigation seemed aware of routine oral hygiene maintenance as nearly 80% participants in all groups reported to brush twice daily and approximately 70% patients in all groups were performing interproximal flossing at least once daily. It is therefore postulated that the mild tobacco smoking habit, relatively younger age group and routine oral hygiene maintenance may have contributed towards absence of clinical signs of periodontal inflammation. However, by no means can be short history of smoking and waterpipe use can be considered “safe” health wise.

The laboratory-based investigations performed in the present study showed that despite relatively young, short history of smoking and waterpipe usage and age adequate adoption of oral hygiene maintenance measures, levels of AGEs were nearly 3 times higher in the GCF of cigarette-smokers and waterpipe-users (with no difference between these groups) compared with non-smokers (Table 3). This reflects that on a molecular level periodontal inflammation is intensifying even though clinical and radiographic evaluations did not demonstrate an active sign of periodontal destruction. These factors may also be associated with a lack of correlation between AGEs, periodontal parameters and clinical parameters in the population under investigation (Table 4). Interactions between AGEs and RAGE in periodontal tissues induce a state of oxidative stress that in turn augment the production of proinflammatory cytokines, inhibit osteoblastic activity and induce apoptosis and periodontal fibroblasts [40]. Studies [41–44] have also shown that tobacco-smoking increases the formation and accumulation of AGEs in tissues including human gingival fibroblasts thereby contributing to the progression of periodontal diseases such as periodontitis. It is hypothesized that prolonged use of tobacco-products massively increases GCF AGEs levels and enhance AGEs-RAGE interactions, which clinically present as periodontal soft-tissue damage and alveolar bone destruction. These results suggest that assessment of GCF for inflammatory markers is a use biological tool for assessment of periodontal disease activity in susceptible patient populations (such as patients with periodontitis).

An alarming finding of the present study was that all cigarette-smokers and waterpipe-users that volunteered to participate in the present study were young and were in their late 20 s; and were residents of Kuwait City. Our results support the outcomes of a cross-sectional study by Alali et al. [45] according to which, smoking is more common among males than females in Kuwait and the mean age at smoking initiation was approximately 18 years. The authors of the present study support the results reported by Alali et al. [45]; and emphasize that there is a dire need to implement public health-related policies through which, the general population could be educated the public about the detrimental effects of smoking on oral and general health.

One limitation of the resent study is that total amounts of AGEs in GCF were not assessed in the present investigation. Although AGEs concentrations in relation to GCF volume were reported; it is speculated that assessment of AGEs data in relation to total amount would have provided a more precise estimation of AGEs in the study groups. Moreover, in the present study microbiological assessment of subgingival oral biofilm samples was not performed. The primary reason for this discrepancy is limitations in funding resources that compelled the authors restrict the parameters of investigation. It is anticipated that counts of periodontopathogenic microbes is higher in the subgingival oral biofilm of cigarette-smokers and water-pipe users compared with never-smokers. Moreover, patients with distressed immunity and dual-smokers were excluded. It is hypothesized that levels of AGEs in oral fluids (such as GCF) are higher in immunosuppressed patients and dual smokers thereby making these individuals more susceptible to periodontal damage than systemically healthy tobacco-product users. Further studies are needed to test these hypotheses. The authors of the present study intended to include another group “never-smokers with periodontal diseases” in the present study. However, during the initial patient screening phase most of the never-smokers with periodontal diseases (nearly 39%; data not shown) reported that they had systemic diseases such as DM and CVD. Moreover, never-smokers with periodontal diseases were relatively old (age range 52–79 years) in contrast to individuals that agreed to participate e in the present study (age range 23–32 years). Hence, in order to minimize the risk of age-related bias this group was excluded from the present investigation. However, it is speculated that the GCF levels of AGEs are lower in never-smokers than smokers with periodontal diseases and further studies are needed to test this hypothesis.

## Conclusion

Clinical periodontal status of individuals with a short history of cigarette-smoking and waterpipe-usage may appear similar to never-smokers. On a molecular level, cigarette-smoking and waterpipe-users express raised levels of AGEs than never-smokers that sirens about the ongoing yet latent periodontal inflammatory process.

## Data Availability

The research data and/or materials are not publicly available as the authors did not entail consents to publish this data; however, the data is available from the corresponding author upon reasonable request.
